# Persistent proteinuria among a cohort of Nigerian children with sickle cell anaemia

**DOI:** 10.1371/journal.pone.0347556

**Published:** 2026-04-30

**Authors:** Angela Esohe Adeoti-Lawani, Gabriel Obiorah Ezeh, Elizabeth Eberechi Oyenusi, Chris Imokhuede Esezobor

**Affiliations:** 1 Department of Paediatrics, National Hospital, Abuja, Nigeria; 2 Department of Paediatrics, Faculty of Clinical Sciences, College of Medicine, University of Lagos, Lagos, Nigeria; University of Illinois at Chicago, UNITED STATES OF AMERICA

## Abstract

**Background:**

Persistent proteinuria is an early and modifiable marker of kidney damage. Although Nigeria has the highest burden of sickle cell anaemia (SCA) globally, the prevalence of proteinuria is unknown largely because many of the studies only determined proteinuria in a single urine sample or used less sensitive methods, such as dipstick. Unlike dipstick screening, quantitative urine protein-creatinine ratio allows more accurate detection of low-grade proteinuria.

**Objectives:**

To determine the prevalence and determinants of persistent proteinuria among children with SCA.

**Methods:**

Children with SCA and age and sex-matched controls attending a publicly funded hospital in central Nigeria were consecutively enrolled. Proteinuria was determined at the first visit using the spot urine protein creatinine ratio and repeated at least 3 months afterwards for those with proteinuria. In addition, serum creatinine, full blood count, and fetal hemoglobin were determined at the first visit. Persistent proteinuria was defined as a urine protein-to-creatinine ratio ≥0.2 at both visits over a follow-up period of at least 3 months. Participants were classified based on the presence or absence of persistent proteinuria, and baseline characteristics were compared using within-group distributions for categorical variables with appropriate statistical tests.

**Results:**

One hundred and forty-nine children, each with SCA and controls, were enrolled, respectively. The median (IQR) age of the study participants was 9.0 (6.0) years for both groups, with 65% being males. Persistent proteinuria occurred in 35 (23.5%) children with SCA versus 4 (2.7%) controls (p-value < 0.01). Children with persistent proteinuria had a lower level of fetal hemoglobin. (adjusted OR 0.583, 95% C.I 0.473–0.719).

**Conclusion:**

About a quarter of children with SCA had persistent proteinuria, an early and modifiable marker of chronic kidney disease.

## Introduction

Children who have sickle cell disease (SCD), particularly SCA, experience chronic hemolysis and repeated vascular obstructions, resulting in acute clinical events and long-term organ involvement [1]. Kidney diseases in children with SCA have been recognized as significant contributors to morbidity and mortality [2]. In Nigeria, up to 18.5 to 27.5% of children with SCA have been reported to have kidney disease [3,4].

Screening for kidney disease can be done by simple non-invasive measures, such as the detection of albuminuria or proteinuria using a urine dipstick or by quantitative measurement of urine albumin or protein [5]. Normal urinary protein excretion in healthy children is less than 150 mg/24hrs or less than 4 mg/m^2^/hr [6]. Proteinuria is a urine-protein creatinine ratio of ≥ 0.2 (more than 0.5 for children 6–24 months of age) in a spot urine sample or more than 4 mg/m^2^/hr in a timed urine sample [7,8]. Protein in urine of 1g/m^2^/day or a urine-protein-creatinine ratio of more than 2 is termed nephrotic range proteinuria [6].

Persistent proteinuria is defined as the presence of proteinuria on two or more occasions separated by 1–2 weeks [9,10]. It reflects structural kidney disease, which may progress to chronic kidney insufficiency [9]. Although persistent albuminuria is highly suggestive of glomerular damage, proteinuria provides a more sensitive marker of kidney injury. This is because proteinuria encompasses both glomerular and tubular proteinuria, and in SCD, tubular damage, often driven by repeated ischemia-reperfusion of the tubulointerstitium, may precede glomerular involvement [9,11]. However, albuminuria and proteinuria are more sensitive markers of kidney damage than changes in serum creatinine because they often develop several months to years before the deterioration in glomerular filtration [1,12]. Current chronic disease classification frameworks, including those proposed by Kidney Disease: Improving Global Outcomes (KDIGO), emphasize albuminuria and estimated glomerular filtration rate for staging disease severity. However, total protein excretion may detect both glomerular and tubular injury and may therefore identify early renal involvement in sickle cell anaemia before conventional markers become abnormal. Similar to the general population, it is the presence of persistent albuminuria or proteinuria that has a strong link with kidney damage in children with SCA [9]. This study, therefore, evaluates persistent proteinuria as an early marker of kidney involvement rather than as a substitute for albuminuria-based staging.

Previous Paediatric studies done in Nigeria determined proteinuria at a single visit or used dipstick urinalysis, which may underestimate early renal involvement. Quantitative protein measurement and confirmation of persistence over time improve diagnostic reliability. Because transient proteinuria is common and benign, and dipstick urinalysis is confounded by the degree of dilution of the urine, these studies may not give a reliable burden of kidney disease among children with SCA [3,13]. Globally, only a few studies have characterized persistent proteinuria in children with SCA [14–16]. We performed this study to determine the prevalence and factors associated with persistent proteinuria among a cohort of Nigerian children with SCA.

## Materials and methods

The study was conducted at the National Hospital, Abuja, Nigeria, from May 2022 to January 2023. The National Hospital is a large public-funded tertiary hospital in the nation’s capital city. The study population consisted of children aged 2–16 years attending the sickle cell clinics of the hospital. To provide a comparison group, age and sex-matched children homozygous for hemoglobin A attending the follow-up clinic were also recruited. Evidence of sickle cell anaemia was based on documentary evidence of HbSS in the child’s medical note. For the controls, hemoglobin electrophoresis was done as part of the study. We excluded participants with a known history of kidney disease, diabetes mellitus, or hypertension. However, a structured assessment of prior AKI episodes was not performed. Also, participants with HIV, febrile illness with a temperature greater than 37.5 °C within the prior two weeks, those having their menses, and those with pyuria or nitrite on urinalysis were excluded. Consecutive participants who met the study eligibility criteria were enrolled.

The research was done in accordance with the Declaration of Helsinki. Ethical approval was obtained from the research and ethics committee of the hospital (HREC No: NHA/EC/066/2021) before commencement of the study. Written informed consent was also obtained from the parents and caregivers, while assent was sought from children aged 7 years and above.

### Sample size determination

The minimum sample size for the study was estimated using the formula for the comparison of proportions in two equal-sized groups [17] [19].


$$\text{n}=\text{r}+\text{1}\;\left({\text{p}}^{*}\right)\left(\text{1}-{\text{p}}^{*}\right)\;({\text{Z}}_{\beta }+\text{Z }\frac{\text{a}}{\text{2}}{)}^{2}$$

$$\;(p_{\text{1}}-p_{\text{2}}{)}^{\text{2}}$$

The sample size for the study was 149 for each group of both subjects and controls.

### Data collection

For each study participant, information on demography, past medical history, and clinical features, including the number of hospitalizations and blood transfusions in the last 12 months prior to the study, was collected at the first visit. The socioeconomic status was assessed using Olusanya’s socioeconomic classification scheme. The anthropometry and blood pressure were recorded for each participant, and z-scores were calculated using the WHO AnthroCalc app. A random spot urine sample was analyzed for protein and creatinine for each participant. Participants were followed up for at least 3 months. For those with proteinuria on the first sample, a repeat urine sample was analyzed three months afterwards. In those with SCA, a blood sample was analyzed at the first visit for complete blood count, serum creatinine, and fetal hemoglobin. We used the turbidometric method autoanalyzer to measure urine protein, and the modified Jaffe method to measure both urine and serum creatinine. Proteinuria was quantified using the urine protein-to-creatinine ratio. Quantitative protein measurement was selected to improve detection sensitivity compared with urine dipstick screening. Albuminuria was not measured separately; therefore, the study evaluated total urinary protein rather than albumin-specific excretion. Fetal hemoglobin was measured using high-performance liquid chromatography. Creatinine estimated GFR was calculated for each subject using the original Schwartz formula [20]. Specific biomarkers of haemolysis (e.g., lactate dehydrogenase, bilirubin, reticulocyte count) were not measured. Genetic testing, including APOL1 risk variant analysis, was not performed due to resource limitations.

Proteinuria was defined as a urine protein: creatinine ratio (Pr:Cr) ≥0.2. Persistent proteinuria was defined as urine Pr:Cr ≥ 0.2 at the two visits separated by 3 months interval. Low-grade proteinuria was defined as urine Pr:Cr between 0.2 to 1.0, while nephrotic range proteinuria was defined as urine Pr:Cr greater than 2.

### Data management and analysis

The data were analyzed using IBM SPSS Statistics 25 (IBM, SPSS Inc., Armonk, New York). Results were presented in tables and charts. All continuous variables were tested for normality of distribution. Because they were skewed, continuous variables were summarized using the median and interquartile range. Categorical variables were summarized using percentages. The Mann-Whitney U test was used to compare the numerical variables, while Chi square test was used to compare the categorical variables. Multivariable logistic regression analysis was carried out to determine the predictors of persistent proteinuria among the children with SCA. The level of significance was set at <0.05.

## Results

A total of two hundred and ninety-eight (298) participants were recruited for this study, including 149 with SCA and 149 homozygous for hemoglobin A acted as the controls. The median (IQR) age of the study participants was 9.0 (6.0) years and was similar for both groups. Half (50%) of the participants were aged 11–15 years. There were more males in both groups, with a male-to-female ratio of 1.9:1 (Table 1).

**Table 1:** General characteristics of the study participants.

Variable	Study Population		Total
	Children with SCA n = 149	Control n = 149	n = 298
Age			
2-5 year, n (%)	27(18.1)	26 (17.4)	53 (17.8)
6-10 year, n (%)	60 (40.3)	61 (40.9)	121 (40.6)
11-15 year, n (%)	62 (41.6)	62 (41.6)	124 (41.6)
Age, year^a^	9.0 (6.0)	9.0 (6.0)	
Females, n (%)	52 (34.9%)	52 (34.9%)	104 (34.9%)
Socio-economic class			
Upper, n (%)	129 (86.6)	125 (83.9)	254 (85.2)
Middle, n (%)	19 (12.8)	17 (11.4)	36 (12.1)
Lower, n (%)	1 (0.7)	7 (4.7)	8 (2.7)
Anthropometry			
Weight for age z score	−0.76 (1.50)	0.34 (1.37)	
Height for age z score	−0.50 (1.8)	0.45 (1.78)	
BMI^b^ z score	−0.78 (1.40)	0.30 (1.33)	

^a^values are median (interquartile range); ^b^body mass index

The majority, 93 (62.4%) of the children with SCA in this study were diagnosed between 1 and 5 years of age. In addition, most 123 (82.6%) were hospitalized less than 3 times, and 131 (87.9%) were transfused less than 3 times in the past 12 months (Table 2).

**Table 2:** Clinical characteristics of children with SCA.

Variable	n=149 (%)
**Age at diagnosis (years)**	
Less than 1	34 (22.8)
1-5	93 (62.4)
6-10	20 (13.4)
Greater than 10	2 (1.3)
**No. of hospitalizations (last 12 months)**	
Less than 3	123 (82.6)
3 and above	26 (17.4)
**No. of blood transfusions (last 12 months)**	
Less than 3	131(87.9)
3 and above	18(12.1)

Ninety (60.4%) of the children with SCA had proteinuria at the first visit compared to 25 (16.8%) of the controls. Thirty-five (23.5%) children with SCA had persistent proteinuria compared with 4 (2.7%) children without SCA (p < 0.001). All but one of the children with SCA and persistent proteinuria (97.1%) had low-grade proteinuria; all of the controls with persistent proteinuria had low-grade proteinuria. Because albumin excretion was not measured independently, comparisons between albuminuria and total proteinuria were not performed.

The prevalence of persistent proteinuria among children with SCA was not significantly different among the different ages or sexes (Table 3). Similarly, persistent proteinuria was not significantly different among children belonging to different socioeconomic classes. Sickle cell anaemia severity features, like the number of hospitalizations, number of blood transfusions in the prior 12 months (≥3), total peripheral white cell count, and hematocrit, were not associated with persistent proteinuria. Similarly, the creatinine-based estimated glomerular filtration rate was similar between the children with persistent proteinuria and those without persistent proteinuria (0.600). In contrast, children with persistent proteinuria had lower fetal hemoglobin of 2.6% compared to those without proteinuria (8.7%: p < 0.001).

**Table 3:** factors associated with persistent proteinuria in children with sickle cell anaemia.

Variable	Persistent proteinuria		p-value
	**Yes (n = 35)**	**No (n = 114)**	
Age Groups			0.744
2-5 year, n (%)	8 (22.9)	20 (17.5)	
6-10 year, n (%)	12 (34.3)	45(39.5)	
≥11 year, n (%)	15(42.8)	49 (43.0)	
Female, n (%)	14 (40.0)	38 (33.3)	0.602
Social class			0.067
Upper class, n (%)	25 (71.4)	100 (87.7)	
Middle class, n (%)	9 (25.7)	12 (10.5)	
Lower class, n (%)	1 (2.9)	2 (1.8)	
≥3 hospitalisation last one year, n (%)	10 (28.6)	16 (14.0)	0.084
≥3 blood transfusion last one year, n (%)	9 (25.7)	9 (7.9)	0.662
Systolic blood pressure, mmHg^a^	98.8 (13.3)	97.5 (14.8)	0.630
Diastolic blood pressure, mmHg^a^	54.7 (10.6)	56.5 (10.7)	0.380
White blood cell count, x10^3^/mm^3a^	11.5 (6.2)	10.6 (4.5)	0.544
Hematocrit, %^a^	23 (5.0)	24 (5.0)	0.074
Fetal hemoglobin level, %^a^	2.6 (2.6)	8.7 (6.4)	<0.001
Estimated glomerular filtration rate, mL/min/1.73 m^2^	131 (56)	131 (36.0)	0.600

^a^Values are median (interquartile range); comparisons were made with Mann Whitney U test

Using multivariable logistic regression, we observed that a higher fetal hemoglobin level was associated with lower odds of persistent proteinuria (AOR 0.583, 95% C.I 0.473–0.719) (Table 4). The other factors, such as the number of hospitalizations, the number of blood transfusions, age, gender, blood pressure, white blood cell count, and hematocrit, were not predictors of persistent proteinuria in children with SCA.

**Table 4:** Predictors of persistent proteinuria in children with SCA.

Independent predictors	B	AOR	95% Confidence interval		p-value
**Lower**	**Upper**	
Age, years					
6-10 (vs 2–5)	0.459	1.432	0.397	5.170	0.584
≥11 (vs 2–5)	0.464	1.559	0.423	5.743	0.504
Sex					
Male (vs female)	0.408	1.504	0.503	4.493	0.465
Social class					
Upper (vs Lower)	−0.768	0.464	0.025	8.496	0.605
Middle (vs Lower)	−2.313	0.099	0.040	2.380	0.154
≥3 hospitalizations (vs < 3)	0.136	1.146	0.268	4.895	0.854
≥3 blood transfusions (vs < 3)	0.626	1.869	0.381	9.179	0.660
Blood pressure(mmHg)					
Systolic	−0.020	0.980	0.948	1.013	0.229
Diastolic	0.018	1.018	0.975	1.062	0.415
Fetal hemoglobin, %	−0.540	0.583	0.473	0.719	<0.001*
Hematocrit, %	0.125	1.033	0.952	1.349	0.159
White cell count, 10^3^/ml	0.088	1.093	0.931	1.283	0.672
Estimated GFR, ml/min/1.73m2	0.002	1.002	0.992	1.012	0.746

GFR glomerular filtration rate, OR odds ratio, * statistically significant

## Discussion

The prevalence of persistent proteinuria, an early modifiable risk factor for CKD in children with SCA, has not been fully explored, as most studies, both in Nigeria and globally, have measured proteinuria at a single visit or used less sensitive methods such as dipstick testing.

This study was conducted to characterize the prevalence of persistent proteinuria among a cohort of children with SCA using a more sensitive method, such as urine protein-to-creatinine ratio on two occasions, three months apart.

In summary, we found that about a quarter of children with SCA had persistent proteinuria compared with 2.7% of the controls, although most of the proteinuria was low-grade. Furthermore, children with SCA and persistent proteinuria had lower fetal hemoglobin levels compared to those without proteinuria.

The prevalence rate of persistent proteinuria in the present study (23.5%) is much higher than the persistent proteinuria prevalence of 3.2% reported by Stallworth *et al* [21], 6.2% by Wigfall *et al* [22], 6.2% by Aloni *et al* [23], 6.7% by Anigilaje *et al* [18], and 2.5% by Akubulio *et al* [24] among children with SCA in the USA, Congo, and Nigeria, respectively. The higher prevalence of persistent proteinuria recorded in this study could be because proteinuria was assessed using urine protein-creatinine ratio, and confirmation over time, which improves detection compared with single-sample dipstick screening used in the other studies. The higher prevalence in the present study may also be due to the detection of all forms of proteinuria and not just albuminuria, as was done in other studies [25,26].

Sickle cell anaemia affects both glomerular and tubular function, underlying the need to routinely measure both functions in children with sickle cell anaemia rather than just albuminuria, which is specific for detecting glomerular integrity. Furthermore, the higher prevalence of persistent proteinuria in the present study compared to those done in resource-rich countries may reflect differences in sickle hemoglobin haplotypes in the different regions of the world and the practice of disease-modifying interventions, such as the use of hydroxyurea and chronic transfusion, which is frequently unavailable for children in our present study [22,27]. Other studies, including older participants, have reported a higher prevalence of proteinuria [28,29]. For example, Niss *et al* [28] reported a prevalence of 52% among a study population aged 2–64 years and a median age of 21 years. In their study, they showed that albuminuria was higher with older age, which is in keeping with the chronic complications of SCA that accrue with age. On the other hand, the higher prevalence of persistent proteinuria in children with SCA compared with healthy non-SCA controls is a consistent finding reported by many authors.[18,23]. The higher prevalence in children with SCA is due to the loss of glomerular filtration permselectivity arising from endothelial activation and inflammation induced by sickled red blood cells [15,30,31] Furthermore, repetitive ischemia-reperfusion injury damages the tubulointerstitium and contributes to proteinuria, impairing tubular reabsorption of protein filtered by the glomeruli [15,30,31].

The higher prevalence of persistent proteinuria in children with SCA, though mostly low-grade in the present study, indicates some degree of kidney damage. Apart from being a marker of kidney disease onset, proteinuria is also a marker of kidney disease progression, with faster kidney function deterioration with heavier proteinuria. Not uncommonly, kidney disease is present in 25–33% of adults with sickle cell disease, and end-stage kidney disease accounts for 14–18% of deaths in this cohort [32–34]. Although CKD staging is commonly based on albuminuria and eGFR, persistent total protein excretion may represent early renal injury in sickle cell nephropathy. Quantitative protein screening may therefore help identify at-risk children before conventional CKD thresholds are reached. Our study further supports the practice of routine screening of kidney disease in children with SCA, preferably using more sensitive methods like the urine protein-creatinine ratio. The availability of proven strategies like the use of hydroxyurea and renin-angiotensin system blockade further supports routine screening for proteinuria in those with SCA.

In this study, we found no association between sociodemographic features of the children and persistent proteinuria. Unlike the studies by Gurkan *et al* [35] and Wigfall *et al* [22], We did not demonstrate an association between persistent proteinuria and age. Similarly, we did not observe any difference in the prevalence of persistent proteinuria and sex. This observation, as with age, is not consistently reported in published works involving children, unlike in studies involving adults in the general population and adults with SCA. Although current clinical recommendations support routine screening for albuminuria beginning around 10–11 years of age in children with SCA, our findings demonstrate that measurable urinary protein abnormalities occur across childhood, including younger age groups.

In this study, clinical events such as the number of hospitalizations for crises and blood transfusions in the prior 12 months had no association with the occurrence of persistent proteinuria. Similar to the findings in this study, Kimaro *et al* [36] in Tanzania did not find any association between clinical events like painful crises and frequency of blood transfusion with kidney dysfunction among children with SCA. In contrast, Wigfall *et al* [22] in the USA reported an association between clinical events such as hospitalization and persistent proteinuria among children with SCA. The present study relied on self or caregiver-reported clinical events, such as the number of hospitalizations for crises and the number of blood transfusions in the prior 12 months, unlike the studies by Kimaro *et al* [36] and Wigfall *et al* [22] which retrieved such information from the participants’ medical records.

White blood cell count and hematocrit were not significantly associated with the occurrence of persistent proteinuria in this study. This is in contrast to findings by Bellisario *et al* [37] in Brazil, who reported that persistent albuminuria was associated with high WBC count, and Wigfall *et al* [22] in the USA, who reported that a high leucocyte count was associated with proteinuria.

In contrast to the findings in this study, Alvarez *et al* [38] and Mickie *et al* [27] in the USA reported an association between persistent proteinuria and low hematocrit. Similarly, an association between persistent proteinuria and low hematocrit was reported by Belisario *et al* [37] in Brazil, and Wigfall *et al* [22] in the USA. The observed difference between these studies and the present study could be because of the older age that was screened compared to the present study. Renal disease worsens with age, and anaemia is a marker of disease severity.

Not unexpectedly, we did not find an association between glomerular filtration rate and persistent proteinuria. Rise in serum creatinine (decrease in glomerular filtration rate measured or estimated using serum creatinine) is a late marker of kidney disease because serum creatinine does not begin to rise until about 50–60% of kidney function is lost. With most children having low-grade proteinuria in the present study, a normal glomerular filtration rate is not unusual. This further supports screening for kidney disease using more sensitive markers like proteinuria. This is particularly important in our study population, where children with SCA had lower weight for age and BMI compared to the control group. In this category of children with chronic undernutrition, weight-independent measures of glomerular filtration rate, like serum cystatin C, may be more sensitive [39,40].

There was a significant association between low fetal hemoglobin levels and the occurrence of persistent proteinuria in this study. This is in keeping with studies conducted in the USA by Niss *et al* [28] and in Brazil by Belisario *et al* [37] who reported that persistent albuminuria was associated with low fetal hemoglobin. High fetal hemoglobin level confers a protective impact on the overall severity of sickle cell disease due to its high affinity for oxygen, which prevents sickling of red blood cells, the initiator of the pathogenic mechanism in sickle cell disease. As expected, improving fetal hemoglobin level is a therapeutic target, such as in the administration of hydroxyurea. In resource-limited settings where universal screening for kidney disease may not be possible, those with low fetal hemoglobin identifies a subset that should be prioritized.

## Conclusion

The prevalence of persistent proteinuria was 23.5% in children with SCA, which was higher than 2.7% in the controls. The majority of children with SCA with persistent proteinuria had low-grade proteinuria. There was no association between persistent proteinuria and age, gender, socioeconomic class, past clinical events, systolic and diastolic blood pressure, hematocrit, white blood cell count, and glomerular filtration rate. In the presence of persistent proteinuria, glomerular filtration rate may remain normal in children with SCA. Low fetal hemoglobin level is a predictor of persistent proteinuria in children with SCA.

It is therefore recommended that children with SCA should be screened for kidney disease, preferably using proteinuria rather than glomerular filtration rate, and those children with low fetal hemoglobin levels should be prioritized for proteinuria screening when universal screening is not feasible. Quantitative urine protein testing may be particularly useful in resource-limited settings where early risk stratification is needed. A longer-course study to determine the progression or regression of proteinuria severity in children with SCA will be useful.

### Limitations of the study

A multiple-centre study would have improved the external generalisability of the study findings. Although persistence was assessed over 3 months, a longer longitudinal follow-up would have allowed better characterisation of the proteinuria and long-term renal outcome in the children with SCA. Unmeasured albuminuria limits the characterization of renal injury and may reduce comparability with CKD staging frameworks. Future studies incorporating the urine albumin-to-creatinine ratio would enable more precise characterization of renal injury. Specific haemolytic markers were not assessed. Also, genetic risk factors, including APOL1 variants, were not analysed.

## Supporting information

S1 File Sample size determination. (DOCX)

S2 File Olusanya social classification scheme. (DOCX)

S3 File Plos_Human_Particpants_Research_Checklist_2025. (PDF)
