# Supplementary material for: Persistent proteinuria among a cohort of Nigerian children with sickle cell anaemia
Source: PLoS One. 2026 Apr 30;21(4):e0347556. doi: 10.1371/journal.pone.0347556 (PMC13132187; doi:10.1371/journal.pone.0347556)
Supplement: S1 File — (DOCX) [file pone.0347556.s001.docx]

**SUPPLEMENTAL MATERIAL**

**Sample size determination**

The minimum sample size for the study was estimated using the formula for the comparison of proportions in two equal-sized groups.

n = r + 1 (p^*^) ( 1 - p^*^) ( Zᵦ + Z a/2 )^2^ ………………………………………..[17]

r (p₁ - p₂ )^2^

n = minimum sample size

r = Ratio of control to cases, 1 for an equal number of cases and controls

p^*^ = Average proportion exposed = proportion of exposed cases + proportion of control exposed/2 = (0.067 + 0.004 /2) = 0.0355

Zᵦ = Standard normal variate for power = for 80% power it is 0.84

Z a/2 = Standard normal variate for the level of significance usually set at 1.96 at 5% type 1 error (p<0.05)

p₁ = prevalence of persistent proteinuria in children with SCA = 6.7% as recorded by Anigilaje *et al[18]*

p₂ = prevalence of persistent proteinuria in school children = 0.4% as recorded by Bidemi *et al[19]* persistent proteinuria was defined as the presence of proteinuria on 3 occasions 4 weeks apart.

p₁ - p₂ = difference in proportion expected based on previous studies. p₁ is the proportion in cases (0.067) and p₂ is the proportion in control (0.004).

n = 1 + 1 (0.0355) (1 -0.0355) (0.84 + 1.96)^2^

1 (0.067 – 0.004)^2^

n = 2 x (0.0355) (0.9645) (7.84)

1 0.003969

n = 135.27, which is approximately 135.

However, giving an Attrition rate of 10%, that is 10/100 x 135 = 13.5

135 + 13.5 = 148.5, which is approximately 149

The sample size for the study = 149 for each group of both subjects and controls

Schwartz formula.[20]

eGFR = K x height (cm)

SCr (mg/dl)

eGFR is estimated GFR in ml/min/1.73m^2^

SCr is serum creatinine.

K = 0.33 in preterm and low birth weight infants

K = 0.45 for infants 1 – 52 weeks

K = 0.55 for children 1 – 13 years

K = 0.55 for adolescent females 13 – 18 years

K = 0.70 for adolescent males 13 – 18 years

(1mg/dl = 88.4µmol/l of plasma creatinine)
