# Supplementary material for: Persistent proteinuria among a cohort of Nigerian children with sickle cell anaemia
Source: PLoS One. 2026 Apr 30;21(4):e0347556. doi: 10.1371/journal.pone.0347556 (PMC13132187; doi:10.1371/journal.pone.0347556)
Supplement: S2 File — (DOCX) [file pone.0347556.s002.docx]

**OLUSANYA SOCIO-ECONOMIC SCORE**

The classification of socio-economic scores based on the father’s occupation and the mother’s education by Olusanya et al.

1. Husband (father’s occupation)

Score: 1. Professionals, top civil servants, politicians, and businessmen

2. Middle-level bureaucrats, technicians, skilled artisans, and well-to-do traders.

3. Unskilled workers and those in general whose income would be at or below the national minimum wage.

1. Mother’s educational level

Score: 0 Education up to university level

1. A secondary or tertiary level below the university
2. No formal education or up to primary level only

The socio-economic class of the child’s mother is obtained by adding the scores from A and B above and graded I – V as appropriate, for example:

Score (1) from A plus score (0) from B = social class I

Score (2) from A plus score (0) from B = social class II

Score (2) from A plus score (1) from B = social class III

Score (2) from A plus score (2) from B = social class IV

Score (3) from A plus score (0) from B = social class III

Score (3) from A plus score (1) from B = social class IV

Score (3) from A plus score (2) from B = social class V

High social class – classes 1 and 11

Medium social class – class 111, and Low social class – class IV and V
